# Complete Genome Sequence of a Tomato Mottle Mosaic Virus Isolate from the United States

**DOI:** 10.1128/genomeA.00167-15

**Published:** 2015-04-02

**Authors:** Kornelia Fillmer, Scott Adkins, Patchara Pongam, Tom D’Elia

**Affiliations:** a Department of Biology, Indian River State College, Fort Pierce, Florida, USA; b U.S. Department of Agriculture, Agricultural Research Service, Horticultural Research Laboratory, Fort Pierce, Florida, USA

## Abstract

Tomato mottle mosaic virus was recently reported from the United States following its original description from Mexico as a novel *Tobamovirus* species. We present the first complete genome sequence of a tomato mottle mosaic virus isolate from the United States.

## GENOME ANNOUNCEMENT

Members of the genus *Tobamovirus* in the family *Virgaviridae* have rigid rod-shaped virions that are readily transmitted by plant-to-plant contact and mechanical inoculation and, in some cases, by seeds (1). There are currently 33 species in the genus *Tobamovirus* (http://www.ictvonline.org/virusTaxonomy.asp), which are distributed worldwide and include tobacco mosaic virus (TMV), the first virus of any type or host identified (2). TMV, tomato mosaic virus, pepper mild mottle virus, and tropical soda apple mosaic virus are tobamoviruses that commonly infect tomato, pepper, and other solanaceous crop and weed plants (3, 4). A novel tobamovirus provisionally named tomato mottle mosaic virus (ToMMV) was recently described from Mexico (5) and subsequently reported from the United States (6). Here, we present the first complete genome sequence of a ToMMV isolate from the United States.

A Nicotiana tabacum “Xanthi nc” local lesion-passaged ToMMV isolate, originally collected in 2010 from a commercial tomato field in Florida, USA, was used to mechanically inoculate the systemic host N. tabacum “Xanthi.” Virions were partially purified from systemically infected tissue using a typical tobamovirus protocol (7). ToMMV genomic RNA was extracted from virions using Trizol reagents (Invitrogen, Carlsbad, CA, USA). A cDNA library was prepared using Ion Total RNA-Seq version 2 and Ion Xpress RNA-Seq bar code kits (Life Technologies, Carlsbad, CA, USA) and subsequently sequenced using an Ion PGM Template OT2 200 kit with an Ion 314 chip on an Ion Torrent Personal Genome Machine (Life Technologies) following the manufacturers’ protocols. Individual reads were mapped using the original ToMMV genome sequence from Mexico (KF477193) as a scaffold with the Torrent Mapping Alignment Program (TMAP version 4.21) for Ion Torrent (Thermo, Fisher Scientific, Waltham, MA, USA). Geneious versus R8 version 8.0.4 (Biomatters, Auckland, New Zealand) and Integrative Genomics Viewer (IGV version 2.3) (8, 9) were used to obtain a complete consensus sequence.

Comparative analysis showed that the U.S. ToMMV isolate sequence (6,399 nucleotides) was 99% identical to the Mexican ToMMV isolate sequence with 27 single nucleotide differences and one insertion (G at 6212). Of the 24 nucleotide differences within the predicted four open reading frames (126- and 183-kDa replication proteins, movement protein, and coat protein) typical of tobamoviruses, 15 were silent, whereas 9 led to an amino acid change. Partial Sanger sequences of this and a second ToMMV isolate from the United States confirmed the insertion at 6212.

### Nucleotide sequence accession number.

The complete genome of the U.S. ToMMV isolate 10-100 was deposited in GenBank under the accession number KP202857.
